# Paternal effects on fetal programming

**DOI:** 10.1590/1984-3143-AR2023-0076

**Published:** 2023-08-28

**Authors:** Carl Robertson Dahlen, Samat Amat, Joel S. Caton, Matthew S. Crouse, Wellison Jarles Da Silva Diniz, Lawrence P. Reynolds

**Affiliations:** 1 Center for Nutrition and Pregnancy and Department of Animal Sciences, North Dakota State University, Fargo, ND, United States; 2 Department of Microbiological Sciences, North Dakota State University, Fargo, ND, United States; 3 U.S. Meat Animal Research Center, Agricultural Research Service, U.S. Department of Agriculture, Clay Center, NE, United States; 4 Department of Animal Sciences, Auburn University, Auburn, AL, United States

**Keywords:** fetal programming, sire, epigenetics, offspring outcomes, paternal programming

## Abstract

Paternal programming is the concept that the environmental signals from the sire’s experiences leading up to mating can alter semen and ultimately affect the phenotype of resulting offspring. Potential mechanisms carrying the paternal effects to offspring can be associated with epigenetic signatures (DNA methylation, histone modification and non-coding RNAs), oxidative stress, cytokines, and the seminal microbiome. Several opportunities exist for sperm/semen to be influenced during development; these opportunities are within the testicle, the epididymis, or accessory sex glands. Epigenetic signatures of sperm can be impacted during the pre-natal and pre-pubertal periods, during sexual maturity and with advancing sire age. Sperm are susceptible to alterations as dictated by their developmental stage at the time of the perturbation, and sperm and seminal plasma likely have both dependent and independent effects on offspring. Research using rodent models has revealed that many factors including over/under nutrition, dietary fat, protein, and ingredient composition (e.g., macro- or micronutrients), stress, exercise, and exposure to drugs, alcohol, and endocrine disruptors all elicit paternal programming responses that are evident in offspring phenotype. Research using livestock species has also revealed that sire age, fertility level, plane of nutrition, and heat stress can induce alterations in the epigenetic, oxidative stress, cytokine, and microbiome profiles of sperm and/or seminal plasma. In addition, recent findings in pigs, sheep, and cattle have indicated programming effects in blastocysts post-fertilization with some continuing into post-natal life of the offspring. Our research group is focused on understanding the effects of common management scenarios of plane of nutrition and growth rates in bulls and rams on mechanisms resulting in paternal programming and subsequent offspring outcomes. Understanding the implication of paternal programming is imperative as short-term feeding and management decisions have the potential to impact productivity and profitability of our herds for generations to come.

## Introduction

Developmental programming is the concept whereby environmental factors inflicted on gametes during their development and concepti during their gestation can have subsequent and long-term impacts on post-natal offspring development. Early evidence of developmental programming came from human epidemiological studies (Barker and Clark, 1997) of pregnant women who experienced severe malnutrition during gestation due to the war-time associated food supply shortage, which is often referred to as the “Dutch Hunger Winter” or the “Dutch Famine”. Epidemiological analysis of this particular population of women revealed that, independent of weight at birth, children gestated during this famine had greater incidence of chronic degenerative diseases, altered structure and function of many organs, and increased behavioral abnormalities and mortality (Roseboom, 2019). In addition, male children with light birthweights had greater incidence of death from coronary disease (Godfrey and Barker, 2001). These studies also revealed programming effects that differed by stage of gestation during exposure and sex of the offspring. Subsequent research in livestock models has revealed that maternal nutrition can have impacts on major organ systems in the offspring including the musculoskeletal, digestive, reproductive, immune, endocrine, and central nervous systems (Wu et al., 2006; Reynolds et al., 2010; Dahlen et al., 2021; Hammer et al., 2023; Ibeagha-Awemu and Khatib, 2023; Zhao et al., 2023).

Though extensive research has been conducted regarding the impacts of maternal environment during pregnancy on subsequent offspring outcomes, a growing body of work indicates that the experiences of a sire during spermatogenesis, sperm residence in the epididymis, and development of seminal fluids in the accessory sex glands may be implicated in offspring outcomes (Chan et al., 2020; Lismer and Kimmins, 2023). This concept is termed as paternal programming and there are a variety of situations controlled by livestock herd managers that could result in paternal programming effects. The current review will focus on the proposed mechanisms of action of paternal programming and when and where these mechanisms are being initiated. The potential effect of timing of perturbations on these paternal programming associated mechanisms will also be discussed followed by a brief overview of research in non-livestock species and an update on current livestock research models of paternal programming.

## Potential mechanisms of paternal programming

Epigenetic alterations, oxidative stress, immune responses, and the seminal microbiome are potential contributors to sire-borne effects on developmental programming. Sire environment can influence epigenetic marks of sperm including DNA methylation, histone modifications, and alterations of several types of coding (mRNA) and non-coding RNAs –miRNA, tRFs, piRNA, lncRNA, circRNA (Champroux et al., 2018; Kretschmer and Gapp, 2022).Though extensive demethylation-remethlylation of the sire epigenome occurs in the embryo after fertilization, some sperm-carried epigenetic marks persist after demethylation, including those of imprinted genes, and can be present in the resultant blastocysts (Wu et al., 2020a). Mechanisms exist for sperm to transfer altered chromatin signatures, DNA methylation, and non-coding RNAs to the embryo, but it is unclear whether alterations in phenotype of offspring seen later in life are the result of an altered cascade of post-fertilization gene expression, or owing to the genes maintaining their pre-fertilization epigenetic marks and initiating cascades of gene expression later in life (Lismer and Kimmins, 2023).

Oxidative stress in sperm can occur under conditions of heat stress, obesity, hypertension, insulin resistance, altered dietary ingredients or planes of nutrition, and psychological disorders (Ayad et al., 2022), which likely comes from mitochondrial and enzymatic processes within sperm cells during these times of stress (Aitken, 2020). Oxidative stress can subsequently lead to DNA damage in the sperm. The combination of oxidative stress and DNA damage has the potential to induce paternal programming effects on offspring resulting from damaged sperm that successfully fertilize an oocyte (Aitken, 2020; Billah et al., 2022).

Insemination is followed by an immune response in the female reproductive tract (Marey et al., 2023). The uterus and oviducts of cattle have site-specific pro- and anti-inflammatory responses at different times after insemination (Marey et al., 2020). These immune and inflammatory responses are likely to be essential for fertilization, implantation, and embryo growth and development (Fair, 2015; Yockey and Iwasaki, 2018). In cultured bovine endometrial epithelial cells, seminal plasma originating from high and low fertility bulls resulted in differential release of cytokines (Nongbua et al., 2020). Cytokine profiles of bovine ejaculates can also be influenced by bull nutrition (Harrison et al., 2023). Therefore, the potential exists for a single female to mount differential immune responses to semen depending on the seminal cytokine profile at the time of mating.

Semen also carries with it a rich and diverse microbiota (Koziol et al., 2022; Webb et al., 2023). Bacteria present in semen could be deposited into the oocyte at fertilization, or act indirectly via modulation of the female immune system (Schoenmakers et al., 2019; Luecke et al., 2022). A recent mouse-based study reported that fish oil feeding to male mice for one spermatogenic cycle before breeding resulted in a reduction in lung inflammation and expression of pulmonary pro-inflammatory cytokines in pups (Rumph et al., 2023). These authors also observed that offspring mice born from sires fed fish oil during spermatogenesis had distinct gut microbiota compared to that of offspring born from control sires, demonstrating the influence of paternal diet on offspring early life microbial colonization (Rumph et al., 2022).

## Where are the effects being initiated?

It’s important to point out that some of the potential programming may originate in the testicle, in the epididymis, in the accessory sex glands during development of seminal plasma that is only added to sperm near the time of ejaculation. Sperm DNA methylation and histone modifications mainly take place within the testicle during early stages of sperm development, with sperm miRNA loading thought to occur in later portions of the tract. However, a portion of the miRNAs present in testicular parenchyma of rams and bulls are also present in segments of the epididymis (Guan et al., 2014; Lima et al., 2021; Wu et al., 2021), indicating that the testicle could have been a loading site for a portion of sperm miRNAs. To further support this concept, an evaluation of non-coding RNAs in bull sperm starting in the testicular parenchyma and continuing throughout the regions of the epididymis revealed dynamic transitions in sperm miRNA content as the sperm moved through the reproductive tract (Sellem et al., 2021). Sperm within the testicular parenchyma contained small amounts of miRNAs, and this amount increased substantially as the sperm moved through the approximately 14 day journey in the epididymis (Sellem et al., 2021).

Extracellular vesicles are released in the epididymis (i.e. epididymosomes) and contain miRNAs which are subsequently loaded into sperm. Once the sncRNA are transferred from the epididymosomes to sperm, they can be delivered to the oocyte at the time of fertilization to regulate maternal RNAs in the cytoplasm of the oocyte, or into the nucleus of the cell to affect the transcriptome of the early embryo (Hur et al., 2017). As proof of concept that epididymosomes are responsible for a portion of offspring programming, several investigators have isolated and injected miRNA from stressed males into unstressed zygotes, which resulted in offspring with a stressed phenotype (Chan et al., 2020; Duffy et al., 2021; Wang et al., 2021).

Interestingly, there is a body of work in rodents comparing vasectomized and intact males that demonstrates specific and independent effects of sperm and of seminal plasma (Morgan et al., 2020; Morgan and Watkins, 2020). In these models, the signaling for altered phenotype is likely originating from components of seminal plasma excreted by accessory sex organs and might include cytokines, hormones, specific nutrients, or extracellular vesicles originating from the ampulla, seminal vesicles, or the prostate glands (Morgan and Watkins, 2020; Roca et al., 2022). An additional concept that cannot be discounted is the potential interaction of the sperm and seminal plasma components with the female reproductive tract in terms of the immune response and extracellular vesicles of uterine origin (Sharma, 2019). Research in livestock species that evaluates the independent and combined effects of sperm and seminal plasma on the female immune response and subsequent offspring programming is warranted.

## Timing of alterations

The timing of sperm or seminal plasma alterations can also be broken into several categories; time in terms of sire age, relative age of developing sperm, lag time from insult to observable effect, and persistence of effects in the epididymis or other regions of the male reproductive tract (Table 1). The effects of sire age can be broken down into further intervals, including the prenatal, pre-pubertal, peri-pubertal, reproductive maturity, and advanced reproductive age periods. Strong evidence in rodent models suggest that stimuli experienced even during the prenatal period can affect epigenetic marks in the offspring’s sperm that are produced during adulthood (Radford et al., 2014). An additional report in sheep indicated that prenatal ewe nutrition can have an impact on offspring epigenetic marks in sperm at reproductive maturity (Toschi et al., 2020). Sperm methylation patterns in post-pubertal bulls are influenced by their respective plane of nutritional management during the first 24 (Perrier et al., 2020), or 32 (Johnson et al., 2022) weeks of age, which also encompasses the period of major Sertoli cell proliferation and the subsequent early increase in the number of germ cells (Negrin et al., unpublished). Perhaps spermatogonial stem cells are particularly susceptible to epigenetic alterations during this early period of rapid proliferation.

**Table 1:** Key concepts related to timing of epigenetic alterations.

Epigenome of sperm in can be impacted prenatally and pre-pubertally
Sperm epigenome changes with advancing sire age
Sperm are susceptible to alterations as dictated by their developmental stage at the time of perturbation
Effects in epididymal-derived signals can be present relatively soon after perturbation and can exist for quite some time

Methylation patterns can be changed with advancing peri-pubertal age. Bulls increasing in age from 10 to 16 months had alterations in their sperm miRNA profiles and DNA methylation patterns (Lambert et al., 2018; Wu et al., 2020b). Once altered during the pre- and peri-pubertal period, some of these DNA methylation patterns persist into adulthood (Gross et al., 2020). Epigenetic alterations continue to change throughout the reproductive lifespan of breeding males, but are especially susceptible to changes during the peri-pubertal and advanced paternal age periods (Ashapkin et al., 2023). The latter could be a mechanism linking advanced paternal age with increased development and health disorders of their children (Phillips et al., 2019).

The time required for spermatogenesis varies among species, with bulls completing the process in roughly 61 days (Staub and Johnson, 2018) and rams every 47 days (Senger, 2012). A critical feature of anatomy to consider at this point is the blood/testis barrier, which comprises tight junctions between Sertoli cells and acts to protect developing sperm cells from blood-borne components of the host system (Xia et al., 2007). The structure of the seminiferous epithelium is such that the youngest developmental stages of sperm are present outside of the blood-testis barrier, and as they advance in developmental age they reposition closer to the lumen of the seminiferous tubule before being released during spermiation. Therefore, the age of developing sperm affects both the physical contact with components outside of the blood-testis barrier (with spermatogonia and sperm stem cells being outside of the blood testis barrier) and the specific type of epigenetic alterations that are able to occur given the dynamic changes in sperm DNA packaging at the different stages of development (Marcho et al., 2020; Kiefer et al., 2021).

Additionally, the nutrient-sensing mTOR pathway can contribute to the regulation of blood-testis barrier and affect sperm at several stages of development (Moreira et al., 2019), providing a potential mechanism through which the sire’s nutrition can impact developing sperm within the testicle. Further evidence of stage of sperm development interacting with external stressors is found in models of bull heat stress recovery, where specific sperm morphological abnormalities can be found in ejaculates at specific times after heat stress (Table 2). This indicates that sperm of different developmental stages respond to stressors differently (Rahman et al., 2018; Garcia-Oliveros et al., 2022).

**Table 2:** Impact of time after heat stress on abnormalities observed in sperm from Nellore bulls.*

**Days after heat stress**	**Sperm location during heat stress**	**Abnormalities observed**
0 to 7	Tail of the epididymis	- Sperm with detached normal heads- Low acrosomal membrane integrity and mitochondrial membrane potential
14 to 42	Undergoing meiosis and spermiogenesis in the testis or residing in body and head of epididymis	- Low sperm motility, plasma membrane integrity, and mitochondrial membrane potential
		- High percentage of major and minor defects and DNA fragmentation
		- Tendency for increased lipid peroxidation
49 to 63	Undergoing mitosis or spermatocytogenesis in testis	- Low mitochondrial membrane potential
		- High percentages of major defects
		- Tendency for increased minor defects
70 to 77	Not differentiated yet, or at earliest stages of spermatogenesis in testis	- Normal motility
		- Intact plasma and acrosomal membranes
		- High mitochondrial membrane potential and integrity of DNA

* Adapted from Garcia-Oliveros et al. (2022).

### Lag from insult to injury

The lag time from a perturbation until an effect manifestation likely depends on the site of the effect (e.g. whether the perturbation affected developing sperm, the miRNA payload in the epididymis, seminal plasma composition, the seminal microbiome, or come combination of these). When male mice were given a single dose of dexamethasone to induce acute stress, relatively few changes were observed in metabolism of offspring sired by sperm collected at 3 hours after dexamethasone administration. However, modest alterations were observed in the offspring sired by sperm collected at 7 days after administration, and many alterations in RNA payload and offspring metabolism were observed from matings with semen collected 14 days after dexamethasone administration (Gapp et al., 2021). In a model of bull heat stress, the miRNA content of extracellular vesicles was altered at 7 days and sperm miRNA was altered at 21 days after heat stress (Alves et al., 2021). These observations were supported by another study where some sperm morphological characteristics were impacted in the period immediately after heat stress and others requiring more time to be present in the ejaculate (Garcia-Oliveros et al., 2022). Samples, however, were not analyzed to characterize the lag time required for altered epigenetic marks to be present in the ejaculate in a bovine model (Garcia-Oliveros et al., 2022).

### Persistence of post-insult effects

The concept of the lag from injury/stressor to effect is also related to the concept of length of time post-insult that the respective effects persist. In a model of a 4-week chronic stress in mice (induced by altering variables including 36 h of constant light, 1 h exposure to predator odor, 15 minute restraint, introducing novel objects or white noise overnight, multiple cage changed, and saturated bedding), untreated sperm were incubated with extracellular vesicles from stressed males at different time points after the stress, and then used to produce in vitro fertilization (IVF) embryos (Chan et al., 2020). Interestingly, the extracellular vesicles collected one week after the stress did not result in altered offspring hypothalamic-pituitary-adrenal (HPA) phenotype, but extracellular vesicles collected 12 weeks after the stress (nearly 2.5 times the duration of spermatogenesis in mice) resulted in an altered HPA phenotype. This observation indicates that effects can persist specifically in extracellular vesicles for some time after the perturbation (Chan et al., 2020). An evaluation of men who were self-reportedly recovering from stress showed large shifts in miRNA expression compared with men who reported being relatively stress-free (Chan et al., 2020). Thus, the persistence of altered RNA payload and potential paternal programming effects in livestock models should be a main target of future investigations.

## Evidence of paternal programming in rodent models

An exhaustive review of rodent models of paternal programming is beyond the scope of this paper. However, it is important to note that many studies have clearly demonstrated that the environment experienced by sires during spermatogenesis impacts offspring phenotype. Paternal programming effects have been observed in offspring of sires managed in over/under nutrition models (Billah et al., 2022), on high fat (Claycombe-Larson et al., 2020) or low protein diets (Watkins et al., 2017). Similar findings were reported in cases of targeted supplementation (folic acid, B-12, methionine; components of the one-carbon metabolism pathway that induces epigenetic alterations; (Lambrot et al., 2013; Bailey et al., 2020), acute and chronic stress (Duffy et al., 2021), exercise (Kusuyama et al., 2020), and exposure to drugs (Toussaint et al., 2023 in press), alcohol (Lee et al., 2013), and endocrine disruptors (Liu et al., 2023).

The array of effects on offspring outcomes is model-dependent. Altered phenotype variables that have been reported include changes in appetite regulation; glucose and insulin metabolism; energy metabolism; growth and development of muscle, fat, and bone; reproductive efficiency; cardiovascular health; temperament; social and cognitive abilities; predator avoidance; drug and alcohol preference and dependency; and propensity for depression-associated behaviors [recently reviewed by Lismer and Kimmins (2023)]. It’s also worth noting that several models have resulted in similar measurable responses in offspring (i.e. alterations in glucose/insulin metabolic responses) (Sharma, 2019), begging the question whether different types of stressors in livestock sires could culminate in similar offspring outcomes. In addition, there are differences in the RNA landscape between rodents and livestock species, thus emphasizing the need for livestock-specific work (Chukrallah et al., 2021).

## Evidence of paternal programming in livestock models

The body of literature in livestock species is not nearly as extensive as in rodent models. Several models exists where management-related strategies have shown mechanisms of paternal programming in livestock similar to those reported in rodent models and will be reviewed below.

### Effects on mechanisms of action associated with paternal programming

Several authors have focused on discovering biomarkers for fertility-related epigenetic changes in bulls used for artificial insemination (AI), and have reported that sperm methylation (Kropp et al., 2017; Costes et al., 2022), miRNA abundance (Werry et al., 2022), and histone modifications (Kutchy et al., 2018) differ among bulls of high or low fertility. In addition, seminal plasma from high and low fertility bulls affected the cytokine response of cultured cells differently (Nongbua et al., 2020). As previously mentioned, sperm methylation patterns (Lambert et al., 2018) and microRNA expression (Wu et al., 2020b) were influenced by age of bulls during the peri-pubertal period.

Plane of nutrition was reported to influence mechanisms of paternal programming, with underfed rams having differentially expressed miRNAs in testicular tissue (Guan et al., 2015), increased DNA damage in sperm (Guan et al., 2014), as well as altered miRNA, mRNA, and pre-mRNA splicing in sperm compared with overfed rams (Guan et al., 2017). In yearling and mature bulls experiencing body weight fluctuations, sperm methylation was altered (Moura et al., 2022) and semen cytokine profiles were altered by plane of nutrition (Harrison et al., 2023). Heat stress in bulls induced oxidative stress, altered sperm characteristics, miRNA profile of sperm and extracellular vesicles (Alves et al., 2021), and sperm chromatin protamination (Rahman et al., 2011). Though each of the models (and other similar models not referenced in the current review) provide a glimpse of their potential to elicit paternal programming responses, fewer livestock models have followed alterations in sperm through the processes of fertilization, embryo and fetal development, and parturition necessary to verify paternal programming responses.

### Models demonstrating paternal programming effects

Specific examples do exist in swine, sheep, and bovine models in which paternal programming was evident (Table 3). A recent report examining the effects of boar housing revealed that boars housed in enriched crates during pre-breeding spermatogenesis sired more piglets than boars housed in crates without enrichment (Sabei et al., 2023). In addition, piglets sired by pen-raised boars had reduced incidence of skin lesions and an attenuated response to potentially painful stimulus (Sabei et al., 2023), indicating effects on stress/emotional response areas of the brain. Feeding methyl donors (methionine, cysteine, choline, betaine, vitamin B6, folate, and vitamin B12) to boars resulted in offspring that had differential gene expression and DNA methylation, as well as altered back fat (Braunschweig et al., 2012). Similarly, when sibling ram pairs were fed a diet with or without a rumen-protected methionine from weaning to puberty their post-pubertal sperm methylation was altered, and subsequent F1 offspring from rams that received methionine had reduced body weight at puberty and had smaller scrotal circumference than their counterparts from rams that did not receive methionine (Gross et al., 2020).

**Table 3:** Summary of livestock models demonstrating paternal programming effects.

**Species and focus**	**Experimental conditions**	**Effects in embryos/offspring**	**Citation**
Boar nutrition	Received methyl donors vs no methyl donors	Differential gene expression and DNA methylation, as well as altered back fat.	Braunschweig et al., 2012
Boar housing environment	Housed in crates, enriched crates, or pens	More piglets born from boars housed in enriched crates and pens, reduced skin lesions, and altered avoidance behavior to skin pressure stimulus.	Sabei et al., 2023
Ram diet	Pre-pubertal methionine feeding vs control	Male offspring (F1) from rams receiving methionine lighter BW and smaller scrotal circumference at puberty.	Gross et al., 2020
Ram diet	Pre-pubertal methionine feeding vs control; evaluation of F2 and F3 generations	Scrotal circumference phenotype and altered methylation patterns observed in sperm of F1, F2, and F3 generations.	Braz et al., 2022
Bull diet	Fed diets containing Omega 3 (fish oil or flax oil) vs saturated fat control	Enhanced blastocyst rate and altered gene expression of blastocysts.	Kalo et al., 2022
Bull age	Semen collected from same bulls at 10, 12, and 16 months of age, then used for IVF on oocytes recovered from same donor cow for each bull.	Differential gene expression and methylation observed as bulls aged, mainly associated with metabolic pathways.	Wu et al., 2020a
Bull fertility status	Semen from bulls with high vs. low sire conception rate	Differential mRNA transcript abundance and methylation in blastocysts.	Kropp et al., 2017
Bull seminal plasma	Cows receiving seminal plasma at AI or not	Seminal plasma tended to increase conception rates to AI, and increased pregnancy rates to AI in sex sorted semen. Increased birth weights from calves born from sex sorted semen.	Ortiz et al., 2019
Bull seminal plasma	Exposure of heifers to SP of vasectomized bulls before transfer of multiple embryos	Enhanced conceptus length at d 14, altered gene expression of conceptus.	Mateo-Otero et al., 2020

Following up on their observations of altered miRNA and DNA methylation in bulls of different ages, Sirard’s research group reported alterations in methylation patterns and gene expression in oocytes derived from IVF using semen collected from the same bulls at 10, 12, and 16 months of age (Wu et al., 2020a). Interestingly, pathway analysis of the resulting blastocysts indicate that metabolic pathways were affected, which warrants further post-natal evaluation. An additional evaluation of the high/low bull fertility model also revealed changes in mRNA abundance and methylation of resultant blastocysts (Kropp et al., 2017). Feeding omega-3 fatty acids in the form of fish meal or flax meal enhanced blastocyst rate and altered blastocyst gene expression compared with a saturated fatty acid control (Kalo et al., 2022)

Seminal plasma has been targeted as having the potential to enhance fertility in cattle breeding systems (Bromfield, 2016). Though not always successful at increasing pregnancy rates, exposing heifers to seminal plasma from vasectomized bulls before transfer of IVF embryos resulted in enhanced embryo growth and altered conceptus gene expression in cattle receiving multiple IVF embryos (Mateo-Otero et al., 2020). Likewise, increased birth weights of calves born from sex-sorted semen was reported (Ortiz et al., 2019). Thus, seminal plasma-specific effects may be present in cattle, and are likely more important in natural mating where seminal plasma is concentrated in the ejaculate rather than in cases of AI where seminal plasma is diluted with semen extender in preparation for cryopreservation, or when adding an aliquot of seminal plasma at the time of AI. Specific components of bovine seminal plasma that elicit programming responses, however, are yet to be determined.

## Final considerations

Though human epidemiology and rodent models have established solid proof of paternal programming effects, the body of evidence in livestock species is limited. Potential mechanisms, magnitude, timing, and persistence of effects, and whether and to what extent specific paternal programming effects are present in livestock species all warrant further exploration. Our group is focused on common management scenarios of plane of nutrition and growth rates in bulls and rams and their effects on mechanisms responsible for paternal programming with the goal of understanding offspring outcomes. Once we demonstrate the impacts of common scenarios under direct control of our herd and flock managers, however, the work is likely just beginning as longer-term and interactive situations must be explored.

Though previously reported in rodent models (Aiken and Ozanne, 2014), a recent report in sheep highlighted the potential for sire experiences during spermatogenesis to not only impact the F1 generation conceived during the initial breeding, but also to impact successive generations. In their model of feeding rumen protected methionine to prepuberal sheep, Kahtib’s lab revealed that paternal programming effects persisted until at least the F2 generation (Braz et al., 2022), with over 100 methylated cytosines and an altered scrotal circumference phenotype inherited by the F1 and F2 generations. Given the long generation interval of cattle, paternal programming effects would be affecting herds for years into the future. Another consideration paramount to the programming equation is the potential additive and/or interactive effects of paternal environment and experiences leading up to mating with those of the breeding females leading up to conception and during gestation on subsequent offspring outcomes. Taken together, as new information becomes available, our existing paradigms of the short-term nature of management decisions (e.g. how should we feed or manage our bulls today for outcomes in the current breeding season) may be shifted to include a long-term outlook on responses that could impact the productivity and profitability of our herds for generations to come.
